# Long non-coding RNA H19 regulates E2F1 expression by competitively sponging endogenous miR-29a-3p in clear cell renal cell carcinoma

**DOI:** 10.1186/s13578-017-0193-z

**Published:** 2017-12-01

**Authors:** Haowei He, Nana Wang, Xiaoming Yi, Chaopeng Tang, Dong Wang

**Affiliations:** 1 Department of Urology, Jinling Hospital, No.305, Zhongshan East Road, Nanjing, 210002 Jiangsu People’s Republic of China; 2 Department of Anesthesiology, Jinling Hospital, Nanjing, 210002 People’s Republic of China

**Keywords:** lncRNA-H19, miR-29a-3p, E2F1, Competing endogenous RNA, Clear cell renal cell carcinoma

## Abstract

**Background:**

Numerous recent studies indicate that the long non-coding RNAs (lncRNAs) are frequently abnormal expressed and take critical roles in many cancers. Renal cell carcinoma is the secondary malignant tumors in the urinary system and has high mortality and morbidity. Around 80% of RCCs is clear cell renal cell carcinoma (ccRCC) and is characterized by high metastasis and relapse rate. However, the clinical significances of lncRNAs in ccRCC are still unknown.

**Methods:**

The human cancer lncRNA PCR array (Yingbio) was performed to detect the differentially expressed lncRNAs in human ccRCC samples. Real-time PCR (RT-PCR), dual-luciferase assay, RNA binding protein immunoprecipitation (RIP) assay, transwell assay, CCK-8 assay, and western blot were performed to explore the molecular mechanism of lncRNAs in ccRCC cell migration and invasion.

**Results:**

In this study, lncRNA-H19 was high expressed and negatively correlated with miR-29a-3p in ccRCC. By bioinformatics software, dual-luciferase reporter and RIP assays, we verified that miR-29a-3p was identified as a direct target of lncRNA-H19. RT-PCR and western blot demonstrated that down-regulated lncRNA-H19 could affect the expression of miR-29a-3p targeting E2F1 with competitively binding miR-29a-3p. Furthermore, transwell assays indicated that lncRNA-H19 knockdown inhibited cells migration and invasion, but this effect was attenuated by co-transfection of lncRNA-H19 siRNA and miR-29a-3p inhibitor. Over expression of E2F1 could rescue lncRNA-H19 siRNA induced suppression on cell migration and invasion in ccRCC cells.

**Conclusions:**

These results show a possible competing endogenous RNAs regulatory network involving lncRNA-H19 regulates E2F1 expression by competitively sponging endogenous miR-29a-3p in ccRCC. This mechanism may contribute to a better understanding of ccRCC pathogenesis, and lncRNA-H19 may be further considered as a potential therapeutic target for ccRCC intervention.

## Background

Renal cell carcinoma (RCC) is one of the most common urological malignant tumors, which constitutes about 3% of all human cancers [1–4]. With different metastasis and relapse rate, RCC fall into three types: clear cell RCC (ccRCC, 70–80%), papillary RCC (pRCC, 10–15%), and chromophobe RCC (chRCC, 5–10%) [5–8]. Adults aged 60–64 are the most prone to ccRCC, however, only 7% of sporadic ccRCC cases are diagnosed at ages younger than 40 years [9–11]. In the last few years, though many advanced approaches and radiotherapy have been made in surveillance and clinical diagnosis, there are adverse clinical outcomes for patients with metastatic ccRCC after curative resection [12].

The human genome project has demonstrated that more than seventy percent of genome sequences can be transcribed and only two percent of these transcripts may encode protein, while most transcripts are considered to as non-coding RNAs [13, 14]. Long non-coding RNAs (lncRNAs) are a heterogeneous class of endogenous non-coding RNAs longer than 200 nucleotides, which are associated with the post-transcriptional gene regulation and some diverse cancer cell behavior, such as proliferation, metastasis, epithelial-mesenchymal transition, and apoptosis [15–17]. In recent research, several lncRNAs (CADM1-AS1 [18], CCAT2 [19], linc00152 [20], lnc-ZNF180-2 [21], MALAT1 [22], SPRY4-IT1 [23] and TCL6 [24]) have already been linked to the initiation and progression of ccRCC.

LncRNA-H19, a non-coding RNA with 3000 bp length and located at chromosome 11p15.5 locus, which is expressed in the cell nucleus and cytoplasm [25, 26]. LncRNA-H19 acts as an oncogene to be involved in various pathological processes of tumor growth and metastasis [27, 28], including breast cancer [29], bladder cancer [30], ccRCC [31], colorectal cancer [32], gastric cancer [33], head and neck squamous cell carcinoma [34], and oesophageal cancer [35]. The expression of lncRNA-H19 is remarkably increased in these cancer tissues, and over expressed lncRNA-H19 promotes cancer cell proliferation, migration, invasion and metastasis. However, the molecular mechanism by which lncRNA-H19 promotes ccRCC proliferation is unknown.

MicroRNAs (miRNAs) are endogenous non-coding RNAs and regulate gene expression by mRNA degradation and translational repression at the post-transcriptional level [36]. Several studies have found that lncRNAs functions as competing endogenous RNAs (ceRNAs) to sponge miRNAs, affecting expression of miRNA targets [37, 38]. However, lncRNA-H19 whether functions as ceRNA to regulate expression of targets with binding miRNA has not been reported in ccRCC.

In this study, we hypothesized that lncRNA-H19 might promote ccRCC cells migration and invasion through inhibiting the expression of miR-29a-3p. In this study, we first detected the differentially expressed lncRNAs in human ccRCC samples by the human cancer LncRNA PCR array (Yingbio), and then measured the expression of lncRNA-H19 and miR-29a-3p in tumor tissues from ccRCC patients. Furthermore, the underlying mechanism of lncRNA-H19 in the development of ccRCC was analyzed in vitro. This study might provide a better understanding of ccRCC pathogenesis and a potential therapeutic target for ccRCC intervention.

## Methods

### Ethics statement

This study was conducted based on our protocols approved by the Ethical Committee of the Jinling Hospital of Nanjing University Medical School. All patients signed written informed consent documents prior to this study.

### Clinical specimens

Thirty ccRCC tissues and their pair-matched normal cancerous tissues for this study were obtained from the patients diagnosed with ccRCC in Department of Urology, Jinling Hospital (Nanjing, China), between March 2011 and September 2015. Written informed consent was obtained from all patients. All tissue was snap-frozen in liquid nitrogen and stored at − 80 °C until use.

### Cell lines, antibodies, inhibitor and plasmid

The human ccRCC cell line (786-O) was purchased from American Type Culture Collection (ATCC). All cells were maintained in RPMI 1640 medium (Genepharma, Shanghai, China) with 10% fetal bovine serum (Invitrogen) at 37 °C with 5% CO_2_ [4]. The primary antibodies anti-E2F1, anti-GAPDH and the second antibodies anti-rabbit or anti-mouse IgG were purchased from Santa Cruz Biotechnology (Dallas, TX, USA). The powdery inhibitor of miR-29a-3p and negative control was bought from Genepharma (Shanghai, China). For E2F1 over expression vector, the E2F1 coding sequences were amplified and inserted into the pcDNA-3.1 vector (Invitrogen) using the *Xho*I and *Eco*RI restriction sites [39].

### SiRNA transfection

The siRNA sequences for lncRNA-H19 (si-H19, 5′-CCAACAUCAAAGACACCAU dTdT-3′), E2F1 (si-E2F1, 5′-CCUGAUGAAUAUCUGUACUdTdT-3′) and negative control (NC, 5′-AUUUCUUUCAUGUUGUGGGTT-3′) were synthesized by Invitrogen (Shanghai, China). The cells were transfected with siRNA for 48 h using Lipofectamine RNAiMAX (Invitrogen, Shanghai) following the manufacturer’s protocol. The efficiency of knockdown was determined by Real-time PCR (RT-PCR).

### Human cancer LncRNA PCR array

Total RNA from tissues was extracted by using Trizol reagent (Invitrogen, Carlsbad, CA, USA) following its manufacturer’s specification. Six purified RNA samples were then sent to Yingbio (Shanghai, China) for Human Cancer LncRNA PCR Array. The array contained 6 reference genes and 84 lncRNAs that were associated with cancer. The lncRNAs were collected from the most authoritative databases, including Ensembl, manually curated lncRNA literature sources, RefSeq and UCSC known genes. The detailed processes were: firstly, 1 μg of total RNA was converted to cDNA with RT2 First Strand Kit (Qiagen, 330401) after assessing integrity. Secondly, cDNA was diluted to a total of 100 μL with ddH_2_O, and 1 μL of this diluent was used for each primer set in RT2 lncRNA PCR Array (Yingbio, Shanghai, China). Thirdly, RT-PCR for PCR assay was performed using the RT2 SYBR Green Mastermix (Qiagen, 330401). Only one replicate for each sample and one pair of primer for each LncRNA were performed for Human Cancer LncRNA PCR Array. The parameters for RT-PCR detection: 95 °C, 10 min; 95 °C, 15 s, 60 °C, 1 min (40 cycles). LncRNAs expression was compared according to the CT value, and data were processed using 2^−ΔΔCT^ method. Differentially expressed lncRNAs with statistical significance (as determined by two-tailed Student’s t test < 0.05) were identified through volcano plot filtering.

### RNA extraction and real-time PCR

Total RNA from all tissues and cells was extracted with Trizol reagent (Invitrogen, Carlsbad, CA, USA). The first-strand cDNA was synthesized using RT2 First Strand Kit (Qiagen) or NCode TM miRNA First-Strand cDNA Synthesis Kit (Life Technologies), respectively. RT-PCR was performed on ABI Q6 detection system (Applied Biosystems Inc., USA) using Real Time SYBR master mix kit (Qiagen, 330401). The RNA expression levels of lncRNA-H19 and E2F1 were calculated relative to expression of GAPDH, and the expression levels of miR-29a-3p was calculated relative to expression of U6 small nuclear RNA. The primers used in this study were as follows: forward, 5′-ATCGGTGCCTCAGCGTTCGG-3′ and reverse, 5′-CTGTCCTCGCCGTCACACCG-3′ for lncRNA-H19; forward, 5′-CTACGTGA CGTGTCAGGACC-3′ and reverse, 5′-GGTGGGGAAAGGCTGATGAA-3′ for E2F1; forward, 5′-GGTCACCAGGGCTGCT TTA-3′ and reverse, 5′-GGATCTC GCTCCTGGAAGATG-3′ for GAPDH; forward, 5′-GGGTAGCACCATCTGAAAT-3′ and reverse, 5′-CAGTGCGTGTCGTGGAGT-3′ for hsa-miR-29a-3p; forward, 5′-GCTTCGGCAGCACATATACTAAAAT-3′ and reverse, 5′-CGCTTCACGAAT TTGCGTGTCAT-3′ for U6. The data were analyzed by using 2^−ΔΔCT^ method.

### Western blot

Whole cell extracts were completed by being lysed in RIPA lysis buffer with protease inhibitor (Promega). The protein concentration of each sample was quantified with BCA assay kit (Pierce Biotechnology, Inc.). Each sample was run on 15% SDS-PAGE gel at the same protein level, and then transferred to PVDF membranes (Millipore). After blocking with a 5% no-fatty milk solution in TBS with 0.1% Tween20, the membranes were incubated for 12 h at 4 °C with the antibodies at a suitable dilution (1:1000). The membranes were then incubated with HRP-conjugated goat-anti-mouse or goat-anti-rabbit IgG as secondary antibodies for 2 h. The signals were recorded with ECL reagents (Millipore ECL plus kit).

### Construction of reporter plasmids and luciferase assays

The method for constructing reporter plasmids has been published elsewhere [40]. HEK293T cells were transfected with the reporter plasmids with Lipofectamine 2000 (Invitrogen, CA, USA). Use of the dual-luciferase reporter assay system (Promega, Madison, WI, USA) to measure luciferase activity has been published elsewhere [41].

### RNA binding protein immunoprecipitation (RIP) assay

According with the manufacturer’s protocol, RIP assay was performed using the EZ-Magna RIP kit (Millipore, Billerica, MA, USA). 786-O with 85% confluence was scraped off and lysed with the RIP lysis buffer. Whole cell extract was incubated with the RIP buffer containing magnetic beads coated with antihuman argonaute2 (Ago2) antibodies (Millipore), and IgG (Millipore) was used as a negative control (input) [37]. After incubation at 4 °C for 2 h, RT-PCR was performed to detect the enrichment of lncRNA-H19 and miR-29a-3p.

### Transwell assay

786-O was removed and suspended in RPMI-1640 medium, after which 1 × 10^5^ cells were added into transwell inserts with 8 mm per size for 24-well plates, and the lower chamber added with 200 mL medium supplement. With using a cotton swab, the non-migrated cells of upper chamber was removed after migration, and the cotton filters were fixed by used of 4% paraformaldehyde. The ability of cell migratory was measured with hematoxylin and eosin staining [42].

### Cell proliferation (CCK8) assay

Cell proliferation was measured by using a CCK-8 kit (Dojundo, Kumamoto-ken, Japan). 2 × 10^3^ 786-O cells/well was seeded in 96-well plates 24 h before experiencing. After respective treatments, the wells received 100 µL culture medium and 10 µL reaction mixture from CCK8 kit. The plates were incubated for 2 h at 37 °C. Then, cell proliferation was estimated by measuring absorbance at 450 nm [43]. All experiments were repeated three times.

### Statistical analysis

The statistical analyses were performed using SPSS 19.0 (SPSS Inc., Chicago, IL, USA). Data was presented as mean ± standard deviation (SD) from three independent experiments with each measured in triplicate. The expression differences between ccRCC tissues and adjacent non-tumor tissues were analyzed with paired Student’s t test. A value of P < 0.05 was considered to be a statistically significant difference.

## Results

### LncRNA-H19 is over expressed in ccRCC tissues and ccRCC cells

**Fig. 1:**
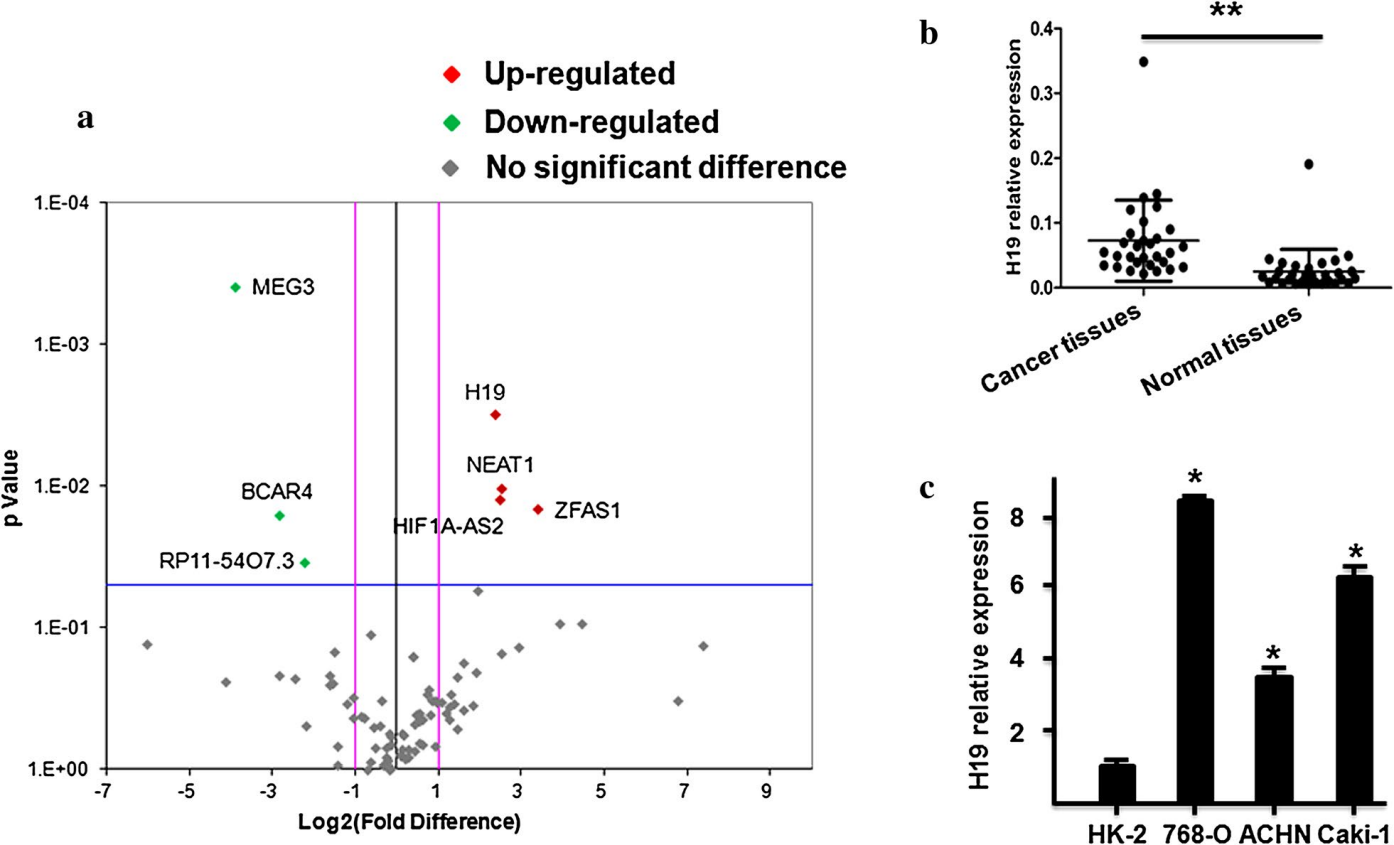
LncRNA-H19 is highly expressed in ccRCC tissues and ccRCC cells. **a** Human lncRNA PCR array for ccRCC tissues. Red indicates an increase and green indicates a decrease in relative mRNA expression. **b** The expression of lncRNA-H19 was normalized to GADPH, differences between ccRCC tissues and normal tissues were compared by Paired Sample T test method (n = 30, P < 0.01). **c** The expression of lncRNA-H19 was normalized to GADPH, differences between HK-2 cells and ccRCC cells (P < 0.05)

To explore for important lncRNAs involved in ccRCC, the lncRNAs expression of ccRCC tissues and their pair-matched noncancerous tissues from three ccRCC patients were detected using the Human Cancer LncRNA Array. A total of 84 lncRNAs were detected, among these, ccRCC tissues contained 4 up expressed, 77 equally expressed, and 3 down expressed lncRNAs compared to their pair-matched noncancerous tissues (Fig. 1a). Compared with noncancerous tissues (Fig. 1a), the expression level of lncRNA-H19 was significantly increased in ccRCC tissues (fold change ≥ 2.0, P ≤ 0.05). Besides, the expression of lncRNA-H19 in 30 paired samples of ccRCC tissues and their corresponding non-carcinoma tissues were determined by RT-PCR. As shown in Fig. 1b, the lncRNA-H19 expression was obviously increased in ccRCC tissues compared to their corresponding normal tissues (P < 0.05). Higher expression levels of lncRNA-H19 were detected in three ccRCC cell lines (768-O, ACHN and Caki-1) than HK-2 cell line (Fig. 1c, P < 0.05).

### Identification of miR-29a-3p as a target of lncRNA-H19

**Fig. 2:**
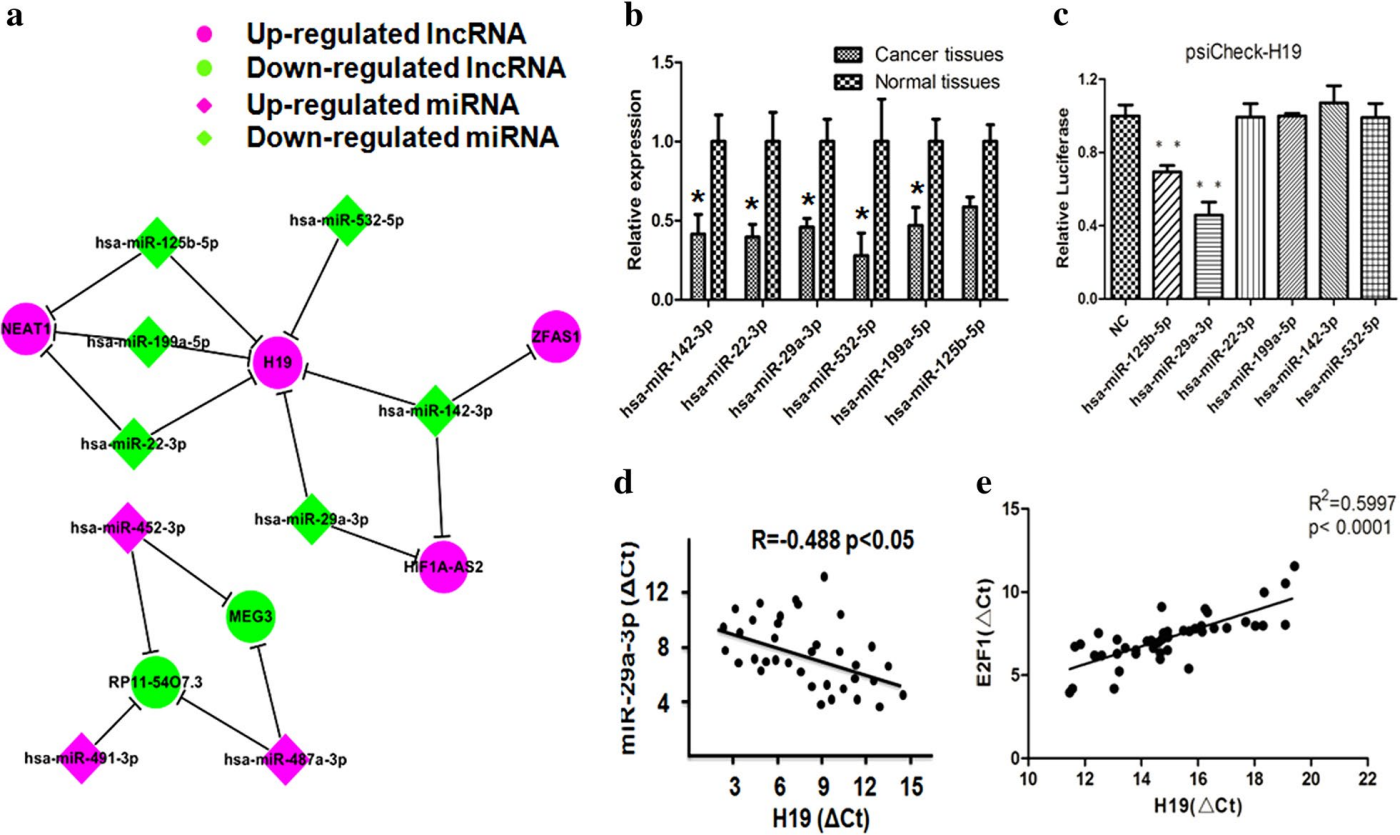
Identification of potential lncRNA-H19 targeting miRNAs. **a** A cohort of six potential miRNAs that could interact with lncRNA-H19 was predicted by starbase 2.0 and Miranda (http://starbase.sysu.edu.cn/ and http://www.microrna.org/). **b** RT-PCR was performed to confirm the interaction between lncRNA-H19 and the six potential miRNAs. **c** Dual-luciferase assay was performed to confirm the interaction between lncRNA-H19 and the six potential miRNAs. **d** Pearson’s correlation was used for correlation analysis between the expression of lncRNA-H19 mRNA and miR-29a-3p mRNA. **e** Pearson’s correlation was used for correlation analysis between the expression of lncRNA-H19 mRNA and E2F1 mRNA

More and more researches have been reported that lncRNAs were identified as ceRNAs for specific miRNAs. To explore whether lncRNA-H19 plays its part through functioning as a ceRNA, we identified the potential lncRNA-H19 targeting miRNAs. With Miranda (http://www.microrna.org/) and starbase (http://starbase.sysu.edu.cn/), six miRNAs (miR-29a-3p, miR-22-3p, miR-199a-5p, miR-532-5p, miR-142-3p, and miR-125b-5p) that could interact with lncRNA-H19 were predicted (Fig. 2a). And then we detected the expression level changes of six miRNAs (miR-29a-3p, miR-22-3p, miR-199a-5p, miR-532-5p, 216 miR-142-3p, and miR-125b-5p) between normal and ccRCC samples by RT-PCR. As shown in Fig. 2b, compared with noncancerous tissues, the expression of miR-29a-3p was significantly decreased in ccRCC tissues. In search of direct target miRNA of lncRNA-H19, dual-luciferase assay was performed. As present in Fig. 2c, our results indicated that miR-29a-3p was probably the downstream target of lncRNA-H19. In addition, the expression of lncRNA-H19 was significant negatively correlation with miR-29a-3p in ccRCC tissues (R = − 0.403, P < 0.05) (Fig. 2d). The expression of lncRNA-H19 was significant positively correlation with E2F1 in ccRCC tissues (R^2^ = 0.5997, P < 0.05) (Fig. 2e).

**Fig. 3:**
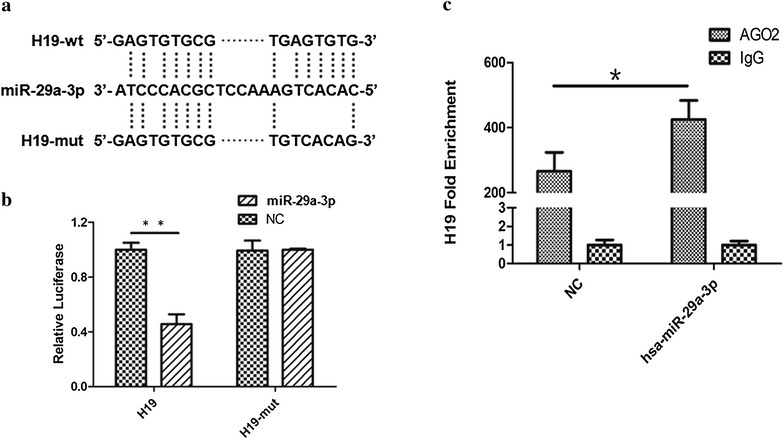
MiR-29a-3p was one target of lncRNA-H19. **a** Representation the lncRNA-H19 and miR-29a-3p binding site by miRanda (http://www.microrna.org/). **b** Dual-luciferase assay was performed to confirm the interaction between lncRNA-H19 and miR-29a-3p. **c** LncRNA-H19 mRNA level was detected in the substrate of RIP assay by RT-PCR. All data were represented as the mean ± SD from three independent experiments, *P < 0.05

To confirm whether miR-29a-3p was targeted and directly bound to lncRNA-H19, the wild-type and mutated fragments of lncRNA-H19 cDNA sequence containing the putative miR-29a-3p recognition site (predicted in starbase 2.0) (Fig. 3a) were cloned. Dual-luciferase assay was performed in HEK293T cells, and then our results indicated that miR-29a-3p mimic significantly decreased the luciferase activities of lncRNA-H19 wild-type fragment (52%) but not lncRNA-H19 mutated fragment (Fig. 3b). These results suggested that miR-29a-3p might have an interaction with lncRNA-H19.

In the cytoplasm, miRNA is one form of miRNA ribonucleoprotein complexes (miRNPs) that also typically contain Ago2 [44, 45]. To test whether lncRNA-H19 associates with miRNPs, RIP assay was performed on 786-O using antibodies against Ago2. As shown in Fig. 3c, lncRNA-H19 was preferentially enriched in Ago2-containing miRNPs relative to control IgG immunoprecipitates. Similarly, miR-29a-3p was detected at a high level greater than that of control anti-IgG. Thus, lncRNA-H19 was present in Ago2-containing miRNPs through association with miRNA-29a-3p, consistent with our bioinformatic analysis and luciferase assays.

### LncRNA-H19 modulated expression of endogenous miR-29a-3p targets E2F1

**Fig. 4:**
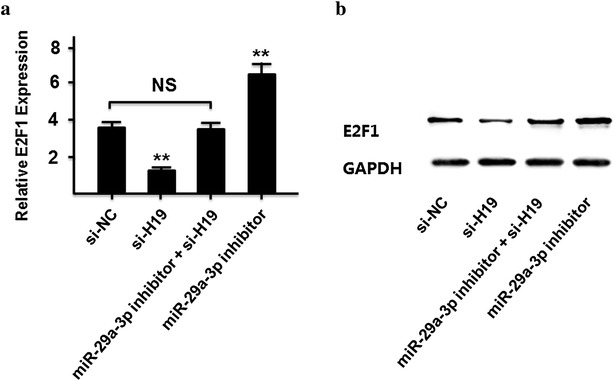
LncRNA-H19 modulated expression of endogenous miR-29a-3p targeting E2F1. **a** The mRNA level of E2F1 in 786-O cells transfected with si-NC, si-H19, miR-29-3p inhibitor, and si-H19 + miR-29-3p inhibitor. **b** The protein level of E2F1 in 786-O cells transfected with si-NC, si-H19, miR-29-3p inhibitor, and si-H19 + miR-29-3p inhibitor

E2F1 was a well known oncogene that is targeted by miR-29a-3p in osteosarcoma [46]. We next measured whether lncRNA-H19 modulated E2F1 expression by targeting miR-29a-3p in 786-O cells. As present in Fig. 4a, b, compared with control group, E2F1 expression at the mRNA and protein levels were down-regulated after lncRNA-H19 silencing and up-regulated by transfecting with miR-29a-3p inhibitors in 786-O cells. However, the modulating effects of lncRNA-H19 on E2F1 were diminished in the group of co-transfecting with miR-29a-3p inhibitor and siRNA-H19.

**Fig. 5:**
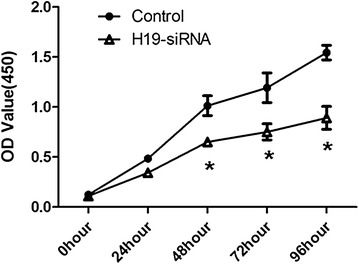
si-H19 inhibited the proliferation of clear cell renal cell carcinoma cells. 786-O cells were plated in a 24-well plate and transfected with si-H19, and cell proliferation was determined at the indicated time points using a CCK-8 assay. All data were represented as the mean ± SD from three independent experiments, *P < 0.05

E2F1 plays a vital role in cell proliferation, it is unclear whether lncRNA H19 has effects on proliferation of clear cell renal cell carcinoma cells. As present in Fig. 5, 786-O cells were plated in a 24-well plate and transfected with si-H19, and cell proliferation was significantly reduced at the different indicated time points using a CCK-8 assay (P < 0.05).

### E2F1 promoted migration and invasion in ccRCC cells

**Fig. 6:**
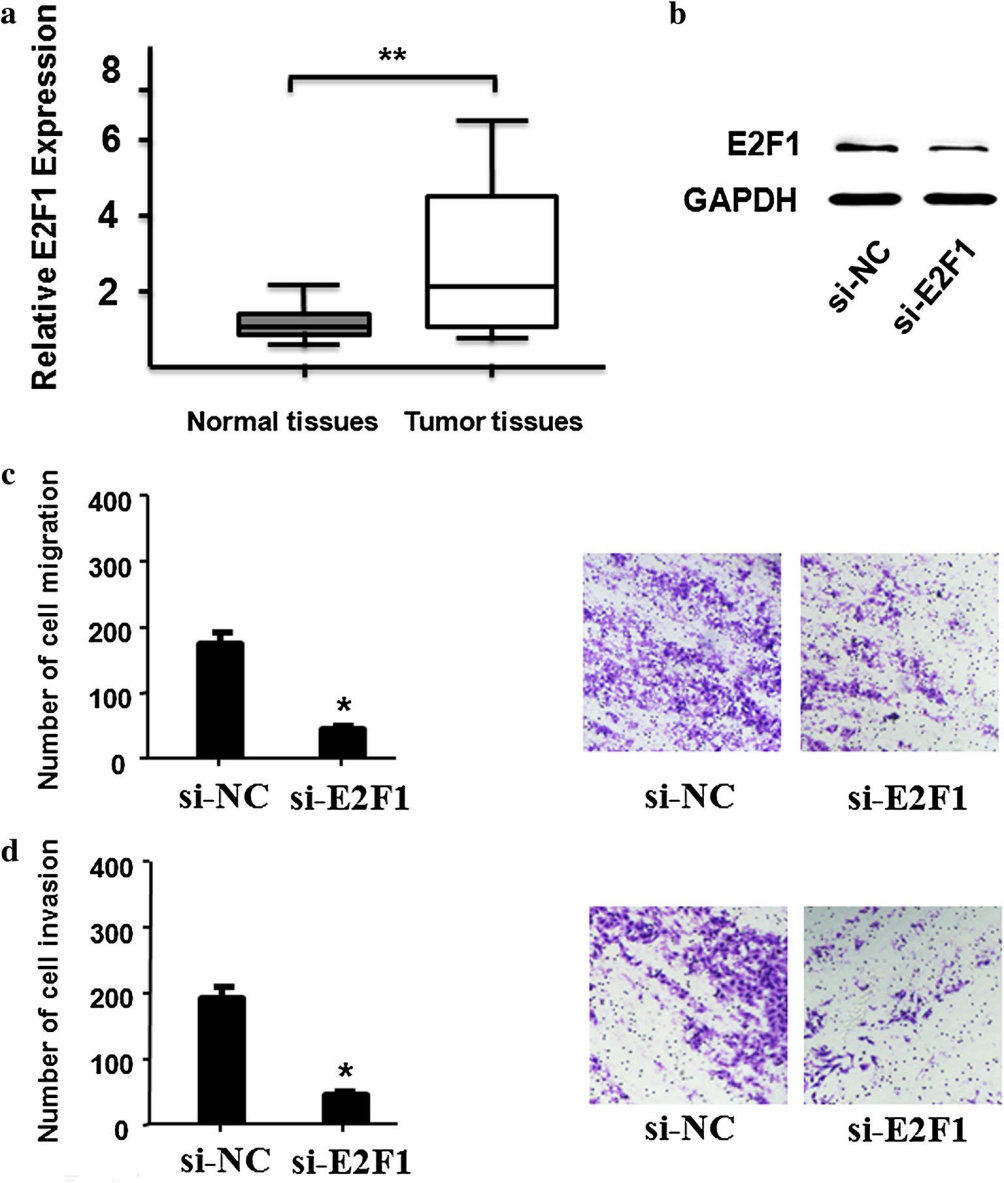
E2F1 promoted cell migration and invasion in ccRCC cells. **a** RNA expression of E2F1 and normal tissues was detected by RT-PCR in ccRCC tissues (n = 30, P < 0.01). The expression of E2F1 was normalized to GADPH. **b** The protein expression of E2F1 after knockdown was detected in ccRCC cells by western blot. **c**, **d** Cells migration and invasion ability and cells number were detected after E2F1 knockdown in ccRCC cells (P < 0.05). All data were represented as the mean ± SD from three independent experiments, **P < 0.01

To investigate the functional roles of E2F1 on ccRCC cells, we measured the expression levels of E2F1 in ccRCC tissues and their matched normal tissues through RT-PCR. Compared with normal tissues, we found that E2F1 was up-regulated in ccRCC tissues (Fig. 6a). Compared to control group, the number of migration and invasive cells was markedly decreased after E2F1 silencing (Fig. 6c, d).

### LncRNA-H19/miR-29a-3p/E2F1 axis on cell migration and invasion in ccRCC cells

**Fig. 7:**
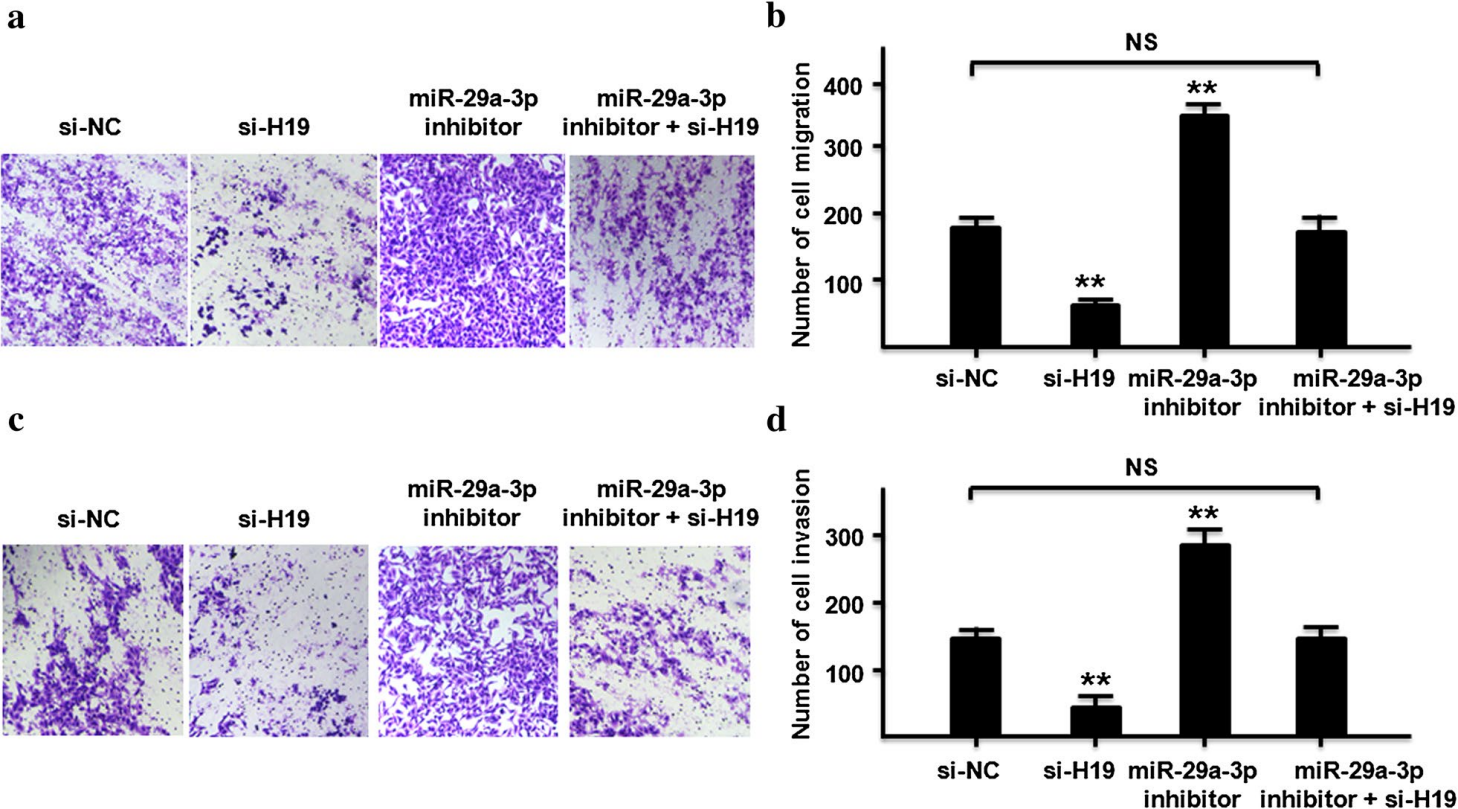
LncRNA-H19/miR-29a-3p/E2F1 axis on cell migration and invasion in 786-O. **a**, **b** Cell migration and cells number were detected by transfecting si-NC, siH19, miR-29a-3p inhibitor, and siH19 + miR-29a-3p inhibitor into 786-O cells (P < 0.01). **c**, **d** Cell invasion ability and invasive cells number were detected by transfecting si-NC, siH19, miR-29a-3p inhibitor, and siH19 + miR-29a-3p inhibitor 786-O cells (P < 0.01). All data were represented as the mean ± SD from three independent experiments, **P < 0.01

**Fig. 8:**
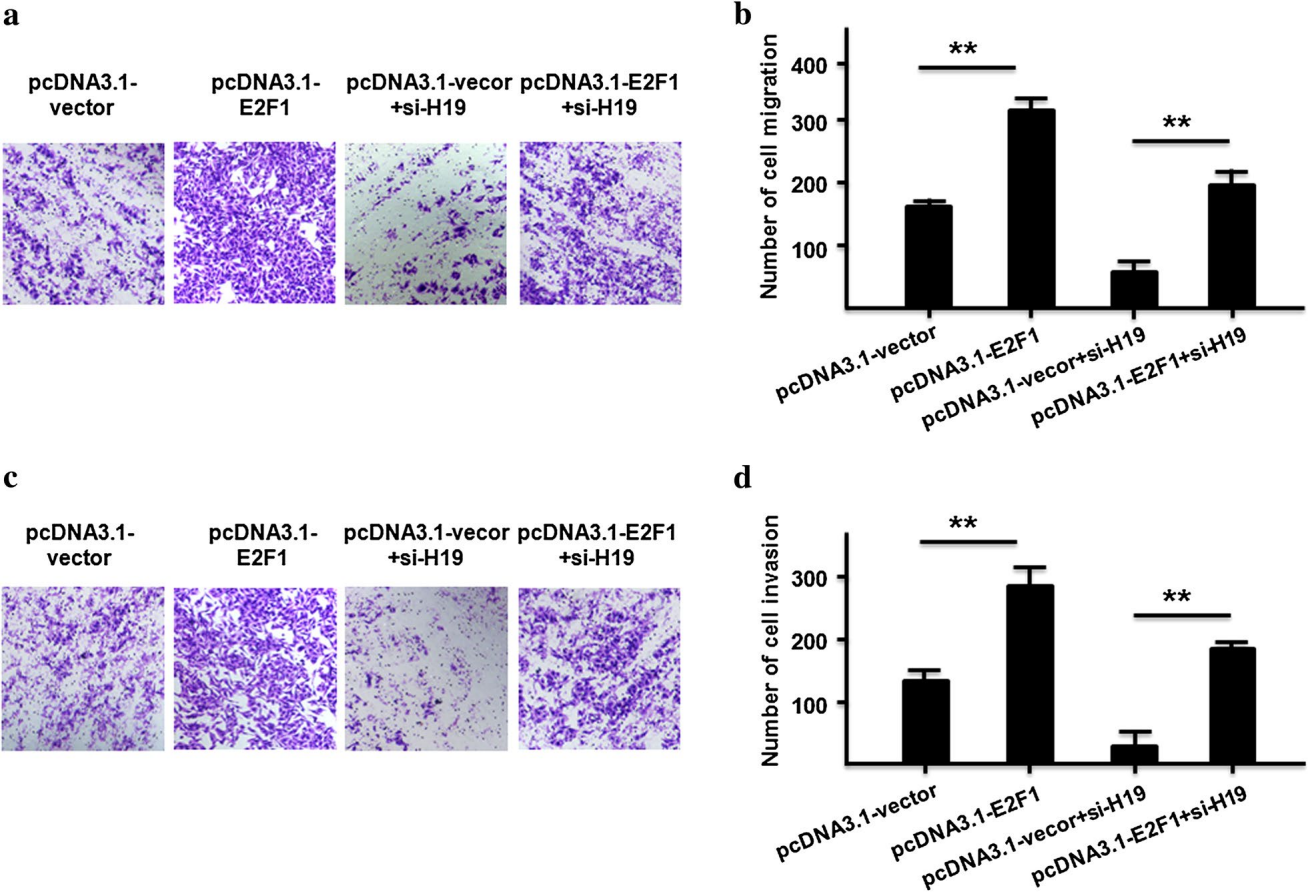
Over-expression of E2F1 rescued H19 siRNA induced suppression on cell migration and invasion in ccRCC cells. **a**, **b** Cell migration and cells number were detected by transfecting pcDNA3.1-vector, pcDNA3.1-E2F1, pcDNA3.1-vector + siH19, and pcDNA3.1-E2F1 + siH19 into 786-O cells (P < 0.01). **c**, **d** Cell invasion ability and invasive cells number were detected by transfecting pcDNA3.1-vector, pcDNA3.1-E2F1, pcDNA3.1-vector + siH19, and pcDNA3.1-E2F1 + siH19 into 786-O cells. All data were represented as the mean ± SD from three independent experiments, **P < 0.01

We continued to explore the effects of lncRNA-H19/miR-29a-3p/E2F1 axis on migration and invasion in ccRCC cells. Initially, compared to control group, knockdown of lncRNA-H19 could decrease the cell number of migration and invasion in 786-O, but was increased by co-transfected with miR-29a-3p inhibitor and siRNA-H19 (Fig. 7a–d). Moreover, we continued to explore that whether over expression of E2F1 could rescue lncRNA-H19 siRNA induced suppression on cell migration and invasion in ccRCC cells. As shown in Fig. 8, compared to control group, knockdown of lncRNA-H19 could decrease the cell number of migration and invasion in 786-O, but was increased by co-transfected with over expression of E2F1 and siRNA-H19 (Fig. 8a–d).

**Fig. 9:**
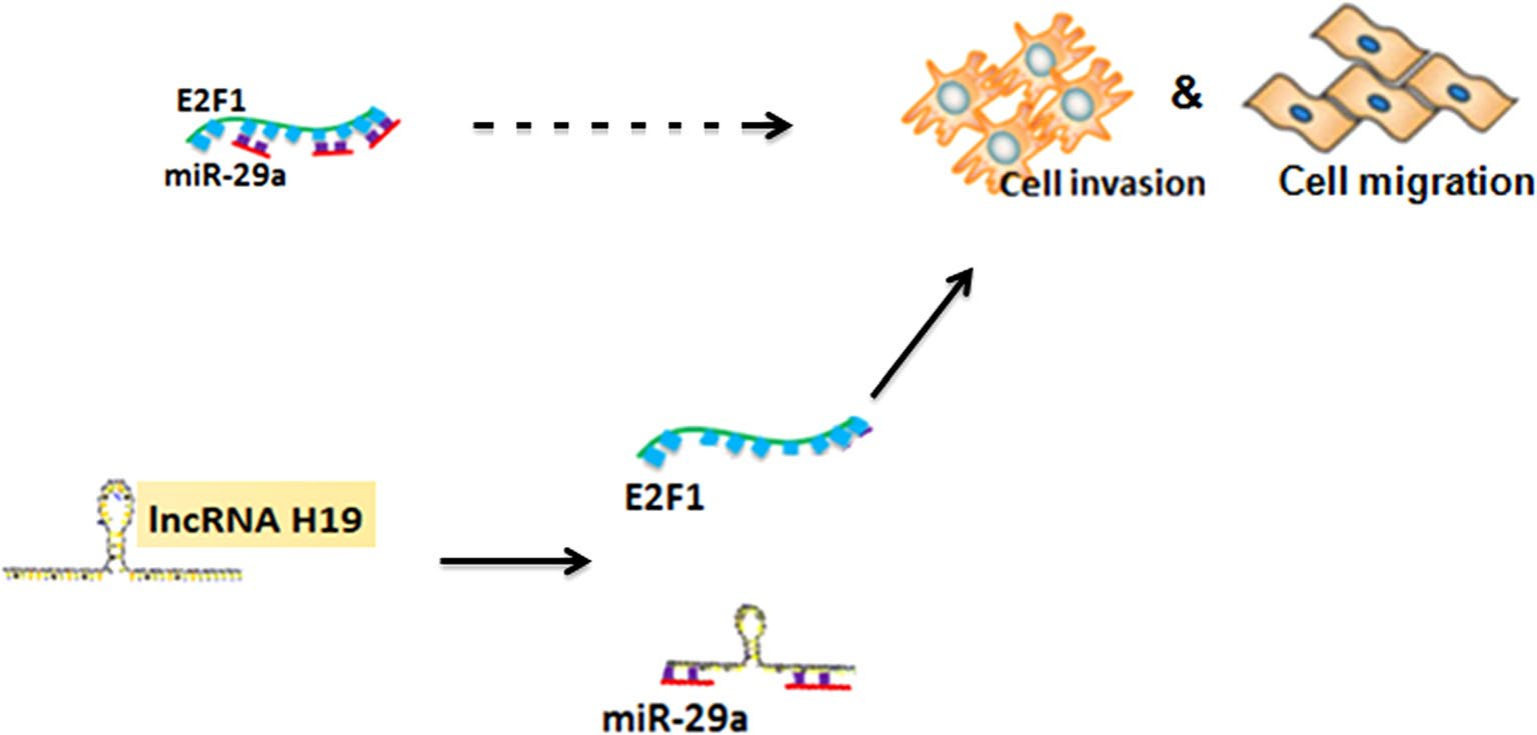
The ceRNA model was proposed to summarize lncRNA-H19/miR-29a-3p/E2F1 pathway. LncRNA-H19 negatively regulated miR-29a-3p in ccRCC, through binding to miR-29a-3p directly. In addition, miR-29a-3p targeted E2F1 and negatively regulated E2F1 expression, indicating that lncRNA-H19 could influence the expression of E2F1 in ccRCC through miR-29a-3p

In conclusion, a potential ceRNA model was proposed to summarize lncRNA-H19/miR-29a-3p/E2F1 pathway (Fig. 9). LncRNA-H19 has the negative effect on the expression of miR-29a-3p in ccRCC through sponging to miR-29a-3p directly. Furthermore, we found that miR-29a-3p targeted E2F1 and negatively affected E2F1 expression, indicating that lncRNA-H19 could influence the expression of E2F1 in ccRCC through miR-29a-3p.

## Discussion

Clear cell renal cell carcinoma is one of the most important human urological diseases in developed and in developing countries. At present, ccRCC patients have a limited option of clinical therapeutic and poor prognosis. Therefore, it is very urgent to study the biological characteristics of ccRCC and find novel molecular markers for cancer prevention and prognosis. In this study, we identified a popular lncRNA-H19 which is over expressed in ccRCC. Our results showed that lncRNA-H19 promotes ccRCC migration and invasion by affecting E2F1 expression with competitively sponging endogenous miR-29a-3p.

LncRNAs are important players in cancer development and emerging in various fundamental biological processes [31–33]. LncRNA HOTAIR is highly expressed in gallbladder cancer tissues and leads to tumor metastases [34, 35]. Linc-POU3F3 is up-regulation in esophageal squamous cell carcinoma samples and promotes tumor development [36]. LncRNA CCAL can increase colorectal cancer cell progression with activating Wnt/β-catenin pathway [38]. In recent studies, lncRNA-H19 revealed two opposite function according to tumor types: one effect of lncRNA-H19 is as an oncogene in breast cancer [37], colorectal cancer [27], glioblastoma [38], and ovarian cancer [36]; the other one effect of lncRNA-H19 is as a tumor suppressor in hepatocellular carcinoma [39], nephroblastoma [41] and prostate cancer [40]. In this present study, we found that lncRNA-H19 functions as an oncogene in ccRCC and promoted cell migration and invasion by up regulating the E2F1 expression in ccRCC cells. Based on this result, our further revealed that lncRNA-H19/miR-29a-3p/E2F1 might be a possible ceRNA regulatory network in ccRCC.

E2F1 is the abbreviation of E2F transcription factor 1, and it encodes the protein which plays a crucial regulation role in controlling cell cycle progression and activating tumor suppressor proteins [47, 48]. Altered expression of E2F1 has been reported in many types of human cancer in conjunction with worse patient survival [49–51]. E2F1 can be used as an effective inducer of cancerous tumor through directly activating transcription of MDM2 and subsequent stimulation of p53-dependent manner [52]. In this study, our results indicated that down regulation of E2F1 might inhibit migration and invasion in ccRCC cells, and the up regulation of lncRNA-H19 was sufficient to increase the expression of E2F1and promote ccRCC migration and invasion. E2F1 plays a vital role in cell proliferation, it is unclear whether lncRNA H19 has effects on proliferation of clear cell renal cell carcinoma cells. In this study, 786-O cells were plated in a 24-well plate, transfected with si-H19 and measured cell proliferation by CCK-8 assay. Our result was consistent with other study [31] and cell proliferation of clear cell renal cell carcinoma cells was significantly reduced at the different indicated time points with si-H19.

It is worth noting that the relationship between lncRNA-H19 and E2F1 depends on miR-29a-3p. Our results proposed that lncRNA-H19 functioned as a ceRNA for miR-29a-3p to regulate E2F1 epigenetically. At the post-transcriptional regulation, lncRNA-H19 could encode the placental specific expression of miR-675 in gestational time point with targeting insulin-like growth factor 1 receptor [53]. Another studies indicated that lncRNA-H19 might work on TGF-β signaling [54] and tumor suppressor RB [55] by miR-675. We found that silenced the expression of lncRNA-H19 could decrease the relative luciferase of miR-29a-3p (Fig. 2c), and the expression of lncRNA-H19 was significant negatively correlation with miR-29a-3p in ccRCC tissues (Fig. 2d). The expression of lncRNA-H19 was not changed by miR-29a-3p mimic (Fig. 3b), which indicated that lncRNA-H19 was the upstream of miR-29a-3p. Our results suggests a potential regulation pathway that involving lncRNA H19 in both molecular and biological aspects in ccRCC.

In this study, we investigated the mechanisms by which lncRNA-H19 exerts its function in the migration and invasion of ccRCC. Our results clearly showed that when silencing lncRNA-H19 expression, ccRCC migration and invasion was inhibited. Our data also found that lncRNA-H19 forms a molecular decoy for miR-29a-3p. MiRNAs are involved in cell proliferation, development, differentiation and metabolism, and play key roles in gene regulation of a number of protein-coding genes. Increasing studies have found that miRNAs can also function by targeting lncRNAs [56, 57]. Hence, lncRNAs may exert their influence on targets by serving as decoys for miRNAs. We also identified E2F1 as an important part of the lncRNA-H19/miR-29a-3p regulatory network. The expression of E2F1 gradually decreased with down regulating the levels of lncRNA-H19.

## Conclusions

In conclusion, in this present study, we aimed to explore the biological function of lncRNA-H19 and its underlying molecular mechanism in ccRCC. Our results indicated that lncRNA-H19 was up-regulated in ccRCC tissues and cells, and was involved in the migration and invasion of ccRCC by regulating miR-29a-3p/E2F1 pathway, implicating the potential application of lncRNA-H19 as an effective prognostic biomarker and novel therapeutic molecular target for ccRCC.

## Abbreviations

LncRNAs: long non-coding RNAs
CcRCC: clear cell renal cell carcinoma
RCC: renal cell carcinoma
PRCC: papillary RCC
ChRCC: chromophobe RCC
MiRNAs: microRNAs
CeRNAs: competing endogenous RNAs
ATCC: American Type Culture Collection
RT-PCR: real-time PCR
SD: standard deviation
